# A Singular and Interesting Case

**Published:** 1887-04

**Authors:** 


### Editorial, Etc.

---

A SINGULAR AND INTERESTING CASE.—A short time ago a German—Gustav M———., aged 55 years, presented himself as a patient at the hospital of the University of Maryland, who claimed that after an attack of yellow fever in New Orleans, in 1853, the saliva gradually ceased to flow from the salivary glands, until his mouth became perfectly dry. Coincident with the suppression of the salivary secretion, his teeth began gradually to crumble away, until they were all destroyed. In 1861, being a soldier, an army surgeon administered to him a remedy which caused a partial return of the salivary secretion as long as it was used. But the nature of the remedy was unknown to the patient, and he was unable to learn its name from the fact that the surgeon who administered it could not afterwards be found by him. The suppression of the saliva has continued from 1861, until the present time, when he presented himself at the hospital. On probing the duct of Steno, a very minute quantity of thick fluid escaped, and on the administration of the fluid extract of jaborandi, saliva was generated to such a degree that he could spit it from his mouth. The digestion of the patient continued good, according to his statement, notwithstanding no saliva was secreted; and the mouth was perfectly dry. The further treatment of this singular case will be regarded with interest.

